# Cerebellar Damage Impairs Executive Control and Monitoring of Movement Generation

**DOI:** 10.1371/journal.pone.0085997

**Published:** 2014-01-17

**Authors:** Emiliano Brunamonti, Francesca R. Chiricozzi, Silvia Clausi, Giusy Olivito, Maria Assunta Giusti, Marco Molinari, Stefano Ferraina, Maria Leggio

**Affiliations:** 1 Department of Physiology and Pharmacology, Sapienza University, Rome, Italy; 2 Department of Psychology, Sapienza University, Rome, Italy; 3 Ataxia Research Lab, IRCCS Santa Lucia Foundation, Rome, Italy; University of Reading, United Kingdom

## Abstract

Executive control of motor responses is a psychological construct of the executive system. Several studies have demonstrated the involvement of the cerebral cortex, basal ganglia, and thalamus in the inhibition of actions and monitoring of performance. The involvement of the cerebellum in cognitive function and its functional interaction with basal ganglia have recently been reported. Based on these findings, we examined the hypothesis of cerebellar involvement in executive control by administering a countermanding task in patients with focal cerebellar damage. The countermanding task requires one to make a movement in response to a ‘go’ signal and to halt it when a ‘stop’ signal is presented. The duration of the go process (reaction time; RT), the duration of the stop process (stop signal reaction time; SSRT), and their relationship, expressed by a psychometric function, are recorded as measures of executive control. All patients had longer go process duration in general and in particular, as a proactive control, as demonstrated by the increase in RT after erroneously performed stop trials. Further, they were defective in the slope of the psychometric function indicating a difficulty on triggering the stop process, although the SSRT did not differ from controls. Notably, their performance was worse when lesions affected deep cerebellar nuclei. Our results support the hypothesis that the cerebellum regulates the executive control of voluntary actions. We speculate that its activity is attributed to specific cerebellar influence over the cortico-striatal loop.

## Introduction

Executive control (EC) allows one to adapt to continuously changing environments by initiating and halting specific actions or monitoring and redirecting the behavior as needed. Improper interactions with the environment, which are associated with several psychiatric and developmental disorders, have been linked to defective EC [1,2]. Several neuroimaging and lesion studies [3,4] have implicated the prefrontal cortex (PFC) as a key node in the cortico-subcortical network that subtends EC with regard to the ability to inhibit actions and monitor performance [5,6].

Of the subcortical structures, the basal ganglia and thalamus have received the most attention [7–10], whereas the cerebellum has been neglected. Recently, using Granger causality and imaging approaches [11], cerebellar output has been proposed to contribute to post-error processing by influencing the activity of the PFC and anterior cingulate cortex (ACC) through connections with the thalamus [12]. Further, the cerebellum could contribute easily to proactive and flexible control of behavior–for example, during post-error adaptation [13,14]–by implementing a forward model to predict the sensory input [15] and influencing the activity of basal ganglia [12], which are implicated in error processing and corrective behavior [16–19].

The contribution of the cerebellum to inhibition is inferred, based on the poor performance of cerebellar patients on the Stroop task [20] and the go/no-go task [21] and reflected by the cerebellar activation that has been observed in imaging studies during the antisaccade task [22] and stop (countermanding) task (CMT) [23]. However, only the behavioral data obtained with the CMT allows one to examine the speed of the stop process and behavioral flexibility following the stop trials and, thus, to determine the function of the cerebellum in motor inhibition and monitoring [24,25]. On the other hand the Stroop task and the go/no-go task, which consider the performance either as the resolution of a conflict between a target and distractor or as the probability of a “nonresponse” following onset of the stimulus, respectively, do not. In support, different mechanisms in the brain for all these tasks have also been implicated by neuroimaging data [5,26].

The aim of this study was to determine the function of the cerebellum in the inhibition of actions and control of performance monitoring by testing patients with focal cerebellar (FCb) damage on a manual CMT. On average, patients reacted to the stop signal as quickly as controls but showed greater variability in triggering the stop process and had longer times in adjusting their behavior after stop failures, particularly when the lesion involved the deep cerebellar nuclei.

## Materials and Methods

### Participants

Thirteen FCb subjects were enrolled; their aetiologies and lesion characteristics are listed in Table 1. The inclusion criteria were no clinical or neuroradiological evidence of extracerebellar pathologies, forming subgroups of similar numerosity with regard to involvement (FCb+; n.7) or noninvolvement (FCb−; n.6) of deep cerebellar nuclei (DCN) in the lesions (as determined by magnetic resonance imaging [MRI]; see below)–the significance of DCN in cognition has recently been highlighted, because they facilitate cerebellar-thalamo-cortical connections [27,28]. The control group comprised 22 subjects, matched for sex, age, and education, with no history of neurological or psychiatric illness. The demographics and education of the groups are shown in Table 2.

**Table 1:** Lesion characteristics in the subjects with focal cerebellar lesions.

Case Code	Side	Aetiology	PICA	AICA	SCA	DCN	ANT	POST	Hem	Vermis	Peduncles
FCb1	R	Surgical	-	-	-	X	X	X	X	X	-
FCb2	L	Ischemic	X	-	-	-	-	X	X	-	-
FCb3	R	Surgical	-	-	-	-	-	X	-	X	-
FCb4	R	Surgical	-	-	-	-	X	X	X	-	-
FCb5	L	Surgical	-	-	-	X	X	X	X	X	-
FCb6	R	Ischemic	X	-	-	X	-	X	X	X	-
FCb7	L	Ischemic	X	-	X	-	X	X	X	X	-
FCb8	R	Ischemic	-	-	-	-	-	-	-	-	X
FCb9	R	Ischemic	X	X	-	X	-	X	X	X	-
FCb10	L	Ischemic	X	-	-	X	X	X	X	X	-
FCb11	L	Ischemic	-	-	X	X	X	X	X	-	-
FCb12	R	Surgical	-	-	-	-	-	X	X	-	-
FCb13	R	Ischemic	-	-	X	X	X	X	-	X	-

Abbreviations: AICA =  anteroinferior cerebellar artery; ANT =  anterior cerebellar lobe; DCN =  deep cerebellar nuclei; Hem =  cerebellar hemisphere; L =  Left, PICA =  posteroinferior cerebellar artery; POST =  posterior cerebellar lobe; R =  right; SCA =  superior cerebellar artery.

**Table 2:** Demographic and intellectual data of cerebellar patients and controls. Means (standard deviation).

Groups	Groups Code	N°	M/F	Age	Education (years)	PM (cut off 18,9)
Control subjects	C	22	10/12	49.1 (11.4)	12.6 (4.7)	31.3 (2.4)
Focal cerebellar lesions	FCb	13	7/6	51.4 (12.5)	13.0 (4.0)	27.6 (3.5)
Lesions involving deep cerebellar nuclei	FCb+	7	6/1	50.8 (12.8)	11.5 (4.7)	29.2 (3.5)
Lesions not involving deep cerebellar nuclei	FCb−	6	1/5	52.1 (13.2)	14.6 (2.5)	25.6 (2.3)
Right side focal cerebellar lesions	FCb_R	8	3/5	47.2 (12.7)	14.2 (3.5)	26.9 (3.1)
Left side focal cerebellar lesions	FCb_L	5	4/1	58.2 (9.6)	11.0 (4.4)	28.6 (4.2)

PM: Raven's 47 (progressive matrices); t-test for independent samples confirmed that FCb patients and controls were well matched for age (p = 0.93) and education (p = 0.73).

Patients' cognitive profiles were evaluated extensively using a battery of tests (Table 3), including WAISr total intelligence quotient values (TIQ), immediate (IR) and delayed (DR) recall of Rey's 15 words [29], immediate visual memory (IVM) [30], forward (FDS) and backward digit span (BDS), forward (FC) and backward (BC) Corsi Test [31], Raven's 47 progressive matrices (PM) [32], freehand copying of drawings (CD) [33], temporal rules induction [34], and word fluency (WF) [35]. FCb patients had generally preserved cognitive patterns. The lack of clinical deficits is consistent with studies that have reported cognitive impairments only under ad hoc test conditions [36–41].

**Table 3:** Neuropsychological assessment. Mean values (standard deviation).

Groups	Tests
	IR	DR	IVM	WF	CD	FDS	BDS	FC	BC	TIQ WAIS-R
FCb	45.0 (10.3)	9.8 (2.5)	19.1 (2.0)	36.2 (13.8)	9.7 (1.7)	6.2 (1.0)	4.3 (1.2)	5.3 (1.1)	4.1 (0.6)	98.6 (13.0)
FCb+	50.0 (5.9)	10.8 (1.6)	19.1 (2.5)	37.5 (11.1)	9.5 (1.8)	6.4 (1.3)	4.2 (1.3)	5.7 (1.3)	4.2 (0.4)	99.8 (16.4)
FCb−	39.2 (11.7)	8.7 (3.1)	19.0 (1.5)	34.8 (17.4)	9.9 (1.6)	6 (0.6)	4.5 (1.2)	4.8 (0.7)	4.0 (0.8)	97.3 (8.9)
FCb_R	44.8 (7.8)	10.1 (2.1)	18.6 (1.3)	36.2 (15.9)	8.9 (1.7)	6.4 (1.3)	4.7 (1.4)	5.2 (1.4)	4.1 (0.6)	101.8 (11.9)
FCb_L	45.3 (14.6)	9.4 (3.4)	19.8 (2.9)	36.2 (11.3)	10.9 (0.8)	6 (0.7)	3.8 (0.4)	5.4 (0.5)	4.2 (0.8)	93.6 (12.9)
Cut off	28.5	4.6	13.8	17.3	7.1	5	3	5	3	100 (15)

IR: Rey's 15 mots short term (immediate recall); DR: Rey's 15 mots long term (delayed recall); IVM: immediate visual memory; WF: word fluency; CD: copying drawings; FDS: forward digit span; BDS: backward digit span; FC: forward Corsi; BC: backwords Corsi; TIQ WAIS – R: Wechsler Adult Intelligence Scale revised.

Motor impairment was quantified in patients using a modified version of the cerebellar motor deficit scale (Motor score) proposed by Apollonio and colleagues [42], which ranges from 0 (absence of any deficit) to 42 (presence of all deficits to the highest degree) and evaluates eight clinical signs (dysarthria, limb tone, postural tremor, upper and lower limb ataxia, standing balance, gait ataxia and ocular movements).

The experimental procedures were approved by the ethical committee of IRCCS Santa Lucia Foundation (CE-PROG.2-AG4-187); written consent for anonymous use of clinical data was obtained from each subject.

### MRI data analysis

T2-weighted scans and 3D-T1-weighted magnetization-prepared rapid-acquisition gradient echo (3D MPRAGE; [43]) (TR = 11.0 ms; TE = 4.4 ms, TI = 300 ms; flip angle = 15°) images were obtained from all FCb subjects using a system that was operated at 1.5 T (Siemens Magnetom Vision, Germany).

Cerebellar lesions were first drawn on each patient's 3D MPRAGE images in native space using MRIcro (www.sph.sc.edu/comd/rorden/mricro.html), thus creating a region of interest (ROI) for each lesion and a corresponding lesion mask, smoothed to 8 mm full width at half maximum (FWHM) and a 0.001% threshold. The 3D MPRAGE images were processed using SPM2 (www.fil.ion.ucl.ac.uk/spm). For each FCb subject, images were reoriented manually according to the default template in SPM2 and normalized into the standard proportional stereotaxic space [Montreal Neurological Institute (MNI)] [44].

**Figure 1:**
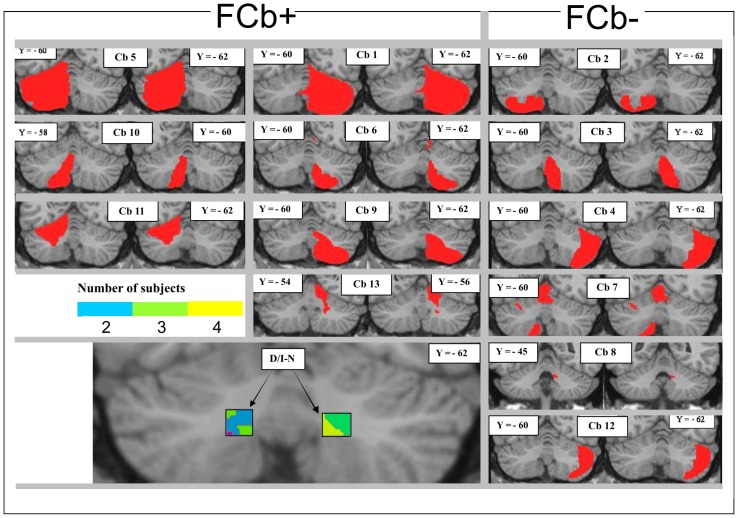
Lesion reconstruction in FCb+ and FCb− patients. Each individual lesion is presented as an overlay of 2 representative coronal MRI T1-weighted templates in stereotaxic space after spatial normalization. The bottom left of the figure shows lesion overlap between patients and a reconstruction of the 3-D probabilistic map (51% to 60%) of right and left dentate/interposed (D/I-N) cerebellar nuclei. The color code indicates the number of subjects whose lesions overlapped with D/I-N nuclei.

The macroscopic cerebellar damage in each patient was assessed after spatial normalization. The involvement of specific cerebellar structures was evaluated with the 3D MRI atlas of the human cerebellum [45] and the 3D MRI atlas of human cerebellar nuclei [46]. To detail the involvement of the nuclei, a 3D probabilistic map (51% to 60%) of right and left dentate/interposed cerebellar nuclei (D/I-N) [45] was reconstructed on a standard template using MRIcro, creating a ROI for the nuclei. Then, the patients' lesions and reconstruction of the nuclei were overlapped (Figure 1).

### Apparatus and task

Participants were seated in a dimly lit room, with their eyes 45 cm from a standard PC monitor (CRT noninterlaced, refresh rate 85 Hz). The presentation of visual stimuli and data acquisition were controlled using Matlab-based routines (www.mathworks.com). A joystick was fixed to the table, aligned with their body midline, and connected to a USB port.

**Figure 2:**
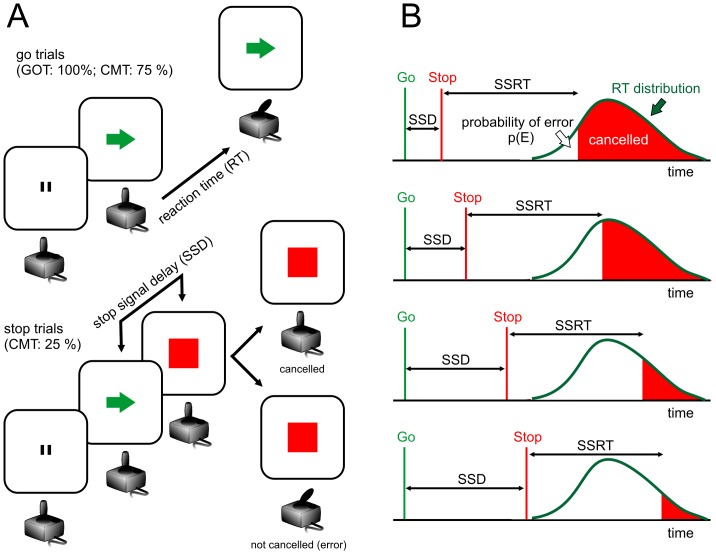
Behavioral tasks and variables. (A) Sequence of events in the go and stop trials for the Go_only (GOT) and countermanding (CMT) tasks. All trials started with the appearance of double vertical bars (fixation point; 2×11 pixels, separated by 2 pixels) in the middle of a screen. After a holding time (800–1200 ms), the central stimulus was replaced by an oriented arrow (Go signal; 12×12 pixels), indicating the direction of the movement. In the go trials (upper part), the subjects had to move the joystick toward the indicated direction as quickly as possible. The time between the Go signal and the onset of movement corresponded to the RT. In the stop trials (lower part), after a variable SSD, a central red square (Stop signal; 200×200 pixels) replaced the arrow, prompting the subjects to withhold the programmed movement and keep the joystick in the resting position. Trials with successfully suppressed movements are indicated as ‘cancelled’; trials with movements executed after a stop signal presentation are indicated as ‘not cancelled’. (B) Relationship among stop signal delay (SSD) duration and probability of error (pE). The longer is the SSD the higher is the pE. By assuming the go process identical in go and stop trials, the reaction time (RT) distribution, and the above relationship could be used to calculate the duration of the stop process (stop signal reaction time; SSRT; see text for details).

Subjects performed 2 versions of a joystick reaction time task (Figure 2A). In the **GO_only task** (GOT), participants moved the joystick in response to a directional go signal (*go trials*). In the **Countermanding task** (CMT), subjects responded to the go signal (CMT *go trials*), as in the GOT, but withheld the movement when, in 25% of the overall trials, a stop signal was presented (*stop trials*) after varying delays (stop signal delay; SSD). In both GOT and CMT the primary task's demand was of responding as quickly as possible to go signal presentation. If subject correctly reacted to the stop signal, the trial was considered canceled, and if the subject failed to stop the movement, the trial was considered not canceled (Figure 2A). When the RT exceeded 1 s in the go trials, the trial was aborted and the subject received an acoustic error feedback. Successfully performed go trials were signaled using different acoustic feedback. Canceled and not canceled stop trials were signaled with 2 acoustic stimuli. Another acoustic stimulus sounded when the RT exceeded 500 ms as a warning for the participant to respect the primary task's demand of responding as quickly as possible.

Each participant performed 2 sessions; one session with the right and one session with the left arm. Each session included 5 blocks of CMT (80 trials each) and 1 block of GOT (60 trials each). The order in which each arm was used was balanced between subjects, and the GOT always preceded the CMT for each arm. To facilitate the subject's performance on the CMT, the starting SSD for each block was always set to the shortest option (17 ms). A rest period (minimum of 2 minutes) between blocks was allowed.

### Behavioral data analysis

A common behavioural finding of the CMT is that the probability of error (pE; i.e., probability of not cancelling the movement) in stop trials increases with the length of the SSD (Figure 2B; white portion of RT distribution). According to the Logan's model [48], the duration of the stop process (stop signal reaction time; SSRT) could be estimated, in each subject, by using the go trials RT distribution and the pE observed for each SSD used. Since is well known that the SSRT estimate is more reliable when obtained with an SSD corresponding to a pE that approximates 0.5 [47], we used a staircase procedure to select the SSD in each stop trial. The SSD was increased by 50 ms after each successfully canceled stop trial and decreased by 50 ms after 2 consecutive not canceled stop trials. The procedure automatically adapts the SSD duration to a subject's performance to obtain a session's pE that approximates 0.5 and is similar between groups (for a similar approach, see [48]).

**Figure 3:**
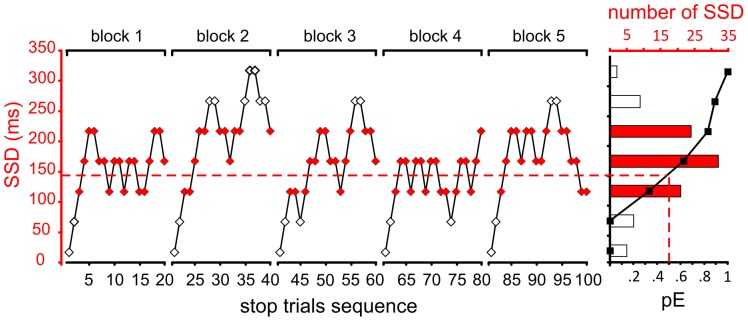
Stop trial sequence and inhibition function. Each block of CMT (for both arms) started with an SSD of 17 ms (1 unit of refresh rate); this delay (stop signal delay; SSD) was increased by 50 ms after each successfully canceled stop trial and decreased by 50 ms after 2 consecutive not canceled stop trials. For each block, red diamonds represent SSDs presented more than 10 times in the session. These SSDs have been used to compute the representative SSD (red interrupted line). Rightmost panel shows the subject's inhibition function (black squares and black solid lines); i.e., the relationship among SSD duration and probability of error (pE). The number of SSDs presented is reported as a histogram. Filled bars indicate SSDs presented more than 10 times.

Figure 3 shows the sequence of consecutive SSDs (red and white diamonds dots) for a CMT session, as adapted by a staircase algorithm to the performance of a control subject in the example. In the right portion of the figure, black squares represent the so-called ‘**inhibition function**’ (i.e., the proportion of not canceled [pE] stop trials at each SSD). The proportion of various SSDs in the session is shown as a histogram, highlighting those that were presented more than 10 times. These SSDs have been used to compute the session's **representative SSD** (braked red line in Figure 3) and to compute the session's SSRT (see [49] for a similar approach). According to the Logan's model [50], the duration of the stop process (SSRT) could be estimated by integrating the CMT *go trials* RT distribution until the integral equals the observed proportion of not canceled trials (pE) corresponding to the representative SSD (integration method; [50]). We also used a second method to estimate the SSRT corresponding to the subtraction of the representative SSD from the CMT RT distribution's average value (mean subtraction method; [50]). The use of at least two methods is common to increase the reliability of SSRT [47,51]. In our study the two methods provided comparable estimates, as confirmed by the analysis reported in the results section. To be consistent with classical approaches, we computed an average value and use it for all comparisons performed.

Before comparing between groups or conditions, the inhibition function obtained for each subject has been normalized as previously described [50] to remove influences due to subject's differences in RT, SSD and SSRT. Thus, for each participant we computed the so-called standardized (z-score) relative finish time (**ZRFT**) [per the equation: ZRFT = (RT_mean_−SSD−SSRT)/RT_SD_, where RT_mean_ and RT_SD_ are the mean and standard deviation, respectively, of the RT in the go trials]. After this normalization, the slope of the inhibition function is believed to measure one's ability to trigger the stop process, regardless of the variability in RT in the go trials, the different values of SSDs used and the estimated value of SSRT [50]. In general, the slope of the inhibition function will be steeper when the inhibitory mechanism is more efficient.

We also computed a measure of the proactive control [52] and the monitoring of the response after each stop signal appearance in the trial sequence or not canceled (error) stop trial. For each subject, we computed **post-stop_correct_** and **post-stop_error_ slowing** by subtracting the mean RT in the go trials after canceled and not canceled stop trials, respectively, from the mean RT in the go trials that were preceded and followed by a go trial (go-go-go sequence).

In FCb patients, we considered the functional arm to be the arm that was contralateral to the compromised cerebellar hemisphere and the hypofunctional arm as the one that was ipsilateral to it. In controls, the dominant and nondominant arms were considered equivalent to the functional and hypofunctional arms, respectively. This approach allows reducing the possibility that patients' impaired performances result statistically significant because of the comparison between controls' dominant arm and patients' affected arm (for a similar approach see [53]). Multifactorial analysis of variance (ANOVA) was used to test the significance of comparisons in RT and SSRT between patients and controls (or between tasks and arm used). Analysis of covariance (ANCOVA) was used to examine differences in the slope of the linear portion (0.15<pE<0.85) of the normalized inhibition function (ZRFT) between groups [54]. Post hoc contrast was conducted using Fisher LSD (for ANOVA) and Tukey-Kramer test (for ANCOVA) with Matlab's multicompare function.

We used an alpha level of 0.05 for all statistical tests. Thus, p values are reported in the text as exact values. However, p = 0.001 also include values of p<0.001. Similarly, when the software does not provide an exact p value, we indicate p<0.05.

## Results

Tables 4 and 5 show the performance of the FCb and Control groups. No significant differences were observed in any measure between arms used. For this reason, the results are reported in the following sections with the arms pooled. We stress, however, that the arm factor was included in all statistical analyses. We will first describe the results for FCb as a homogeneous group and then for the FCb+ and FCb− subgroups separately. Additional analyses were performed with regard to the side of the lesion as the main factor to compare patients with lesions in the right (FCb_R) and left (FCb_L) cerebellum (Table 2).

**Table 4:** Behavioral results. Mean values (standard deviation) of RT in the GOT CMT and post-stop trials slowing.

Groups	RT (GOT)	RT (CMT)	PostC slowing		PostE slowing	
	HF/ND	F/D	HF/ND	F/D	HF/ND	F/D	HF/ND	F/D
Controls	419 (44)	413 (55)	457 (49)	446 (37)	16 (35)	26 (23)	41 (40)	43 (54)
FCb	462 (41)	458 (54)	545 (71)	528 (60)	55 (54)	48 (40)	85 (53)	94 (67)
FCb+	460 (52)	453 (61)	560 (69)	544 (62)	76 (62)	54 (53)	90 (63)	109 (81)
FCb−	466 (41)	458 (54)	545 (71)	528 (60)	30 (37)	41 (41)	79 (43)	76 (47)
FCb_R	460 (48)	454 (58)	461 (79)	539 (68)	64 (66)	61 (44)	93 (83)	84 (84)
FCb_L	467 (46)	464 (51)	521 (54)	510 (46)	40 (30)	29 (25)	94 (35)	87 (42)
Controls (males)	419 (46)	400 (57)	450 (61)	439 (46)	16 (38)	29 (28)	29 (44)	20 (56)
Controls (females)	418 (45)	423 (53)	463 (38)	452 (26)	17 (32)	25 (25)	51 (35)	62 (45)

HF/ND: hypofunctional/nondominant arm; F/D: functional/dominant arm Controls group was sorted for gender differences. Three-way ANOVA (factors: gender, task, arm) revealed no gender effect on GOT or CMT RT for controls (main effect gender: F(1,20) = 1.69, p = 0.21; interaction between task and gender: F(1,20) = 0.002, p = 0.97). Significant difference in arm used was not detected (main effect arm: F(1,20) = 1.75, p = 0.20). Regardless of gender, all control subjects performed with longer RTs on the CMT than on the GOT (main effect task: F(1,20) = 5.0, p = 0.04). Analysis of post-stop go trials in CMT did not reveal gender to be a main effect (F(1,20) = 1.44, p = 0.24) or differences between arms (main effect arm: F(1,20) = 1.61, p = 0.43). PostE was increased in both groups (main effect trial type: F(1,20) = 7.15, p = 0.01) with a significant increase in females (interaction between trial type and gender: F(1,20) = 5.39, p = 0.03 and post hoc test: p = 0.02).

**Table 5:** SSRT estimate.

Group	Estimate	HF/ND	Ttest	F/D	ttest
Control	*SSRT Integration*	*310 (50)*		*304 (41)*	-
	*SSRT mean subtracion*	*294 (53)*	t (42) = 1.02; p = 0.31	*294 (41)*	t (42) = 0.77; p = 0.45
	***SSRT Average***	***302 (51)***		***299 (41)***	
FCb	*SSRT Integration*	*316 (74)*	-	*315 (74)*	-
	*SSRT mean subtracion*	*302 (75)*	t (24) = 0.49; p = 0.63	*306 (65)*	t (24) = 0.30; p = 0.76
	***SSRT Average***	***309 (74)***		***311 (69)***	
FCb −	*SSRT Integration*	*333 (85)*	-	*354 (38)*	-
	*SSRT mean subtracion*	*321 (77)*	t (10) = 0.26; p = 0.78	*340 (24)*	t (10) = 0.73; p = 0.48
	***SSRT Average***	***327 (81)***		***347 (31)***	
FCb +	*SSRT Integration*	*302 (67)*	-	*281(83)*	
	*SSRT mean subtracion*	*286 (75)*	t (12) = 0.42; p = 0.68	*277 (77)*	t (12) = 0.09; p = 0.93
	***SSRT Average***	***293 (71)***		***279 (79)***	
FCb_L	*SSRT Integration*	*350 (50)*	-	*356 (49)*	-
	*SSRT mean subtracion*	*335(54)*	t (8) = 0.46; p = 0.66	*339 (46)*	t (8) = 0.57; p = 0.59
	***SSRT Average***	***343 (51)***		***347 (47)***	
FCb_R	*SSRT Integration*	*295 (82)*	-	*289 (78)*	-
	*SSRT mean subtracion*	*281 (82)*	t (14) = 0.34; p = 0.74	*286 (70)*	t (14) = 0.08; p = 0.94
	***SSRT Average***	***288 (81)***		***287 (73)***	
Controls (males)	*SSRT Integration*	*291 (55)*	-	*295 (26)*	-
	*SSRT mean subtracion*	*273 (64)*	t (18) = 0.69; p = 0.50	*282 (30)*	t (18) = 1.03; p = 0.31
	***SSRT Average***	***283 (59)***		***288 (27)***	
Controls (females)	*SSRT Integration*	*325 (41)*	-	*311(52)*	-
	*SSRT mean subtracion*	*312 (35)*	t (22) = 0.87; p = 0.40	*304 (47)*	t (22) = 0.34; p = 0.74
	***SSRT Average***	***319 (37)***		***308 (49)***	

Mean values (standard deviation). HF/ND: hypofunctional/nondominant arm; F/D: functional/dominant arm. The two methods did not differ in estimating the SSRT for each used arm in each experimental group, and their average was used for SSRT comparisons between groups and arms. T-tests comparisons between the methods are reported. Two-way ANOVA (factors: gender, arm) revealed no gender differences between female and male controls (main effect gender: F(1,20) = 2.56, p = 0.13; main effect arm F(1,20) = 0.10, p = 0.76; interaction between gender and arm: F(1,20) = 1.03, p = 0.37).

### Go process duration and stop process triggering are impaired in patients affected by focal cerebellar lesions

**Figure 4:**
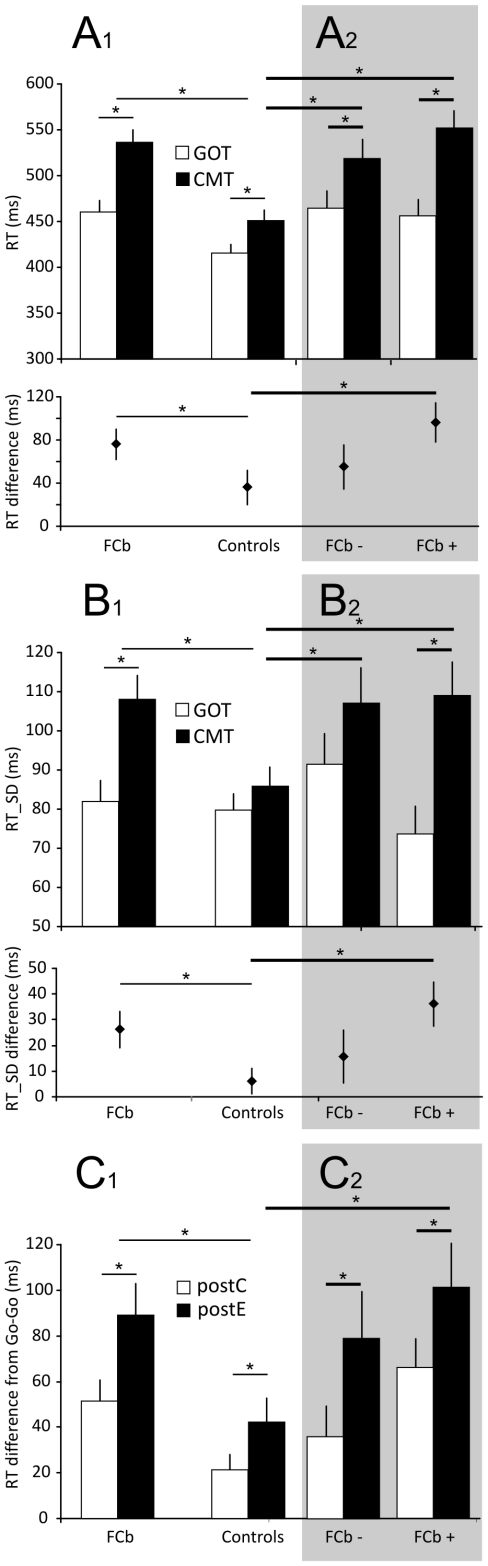
Go process analysis for groups of FCb patients and controls. The left portion of each panel shows data from FCb groups and controls; the right portion of each panel (highlighted by the grey area) shows FCb− and FCb+ patient data. (A) Reaction time in the go trials of the GOT (white bars) and the CMT (black bars) task for FCb patients and controls (A1) and after sorting for DCN involvement in the cerebellar lesion (A2). The lower part of panel A1 and A2 shows the average difference in RT (CMT – GOT) for each of the groups indicated. (B) RT standard deviations (RT_SD) for the same groups as in A. The lower part of panel B1 and B2 shows the average difference in RT_SD (CMT – GOT) for each of the groups indicated. (C) Difference in RT of go trials following stop canceled trials (postC) and go trials following stop not canceled (error) trials (postE) from go trials preceded and followed by a go trial (go-go-go sequence) for the same groups in A and B. Significant differences (p<0.05) between groups and tasks are indicated (*). Thick lines indicate significant comparisons when considering FCb−, FCb+, and controls.

By 3-way (factors: group, task, arm) ANOVA, on the GOT and CMT, FCb patients [factor group; F(1,33) = 28.70; p = 0.001; post hoc: FCb GOT vs control GOT, p = 0.013; FCb CMT vs control CMT, p = 0.001] needed significantly more time to respond (RT) to the go signal than controls (Table 4 and Figure 4 A1). FCb patients, as controls, had a longer RT on the CMT than on the GOT [factor task; F(1,33) = 23.50; p = 0.001; post hoc: FCb GOT vs FCb CMT, p = 0.001; Control GOT vs Control CMT, p = 0.016), suggesting that they adapt their behavior to the increased cognitive demand (presence of stop trials in the block] of the CMT. A significant main effect of the arm factor [F(1,33) = 3.80; p = 0.06] or interactions between it and other factors were not detected [interactions arm × task: F(1,33) = 0.32, p = 0.58; arm × group: F(1,33) = 0.82, p = 0.78; arm × task × group: F(1,33) = 0.31, p = 0.58]). In comparisons with controls, FCb patients slowed their RT in the CMT more than in the GOT. Statistical analysis revealed significant differences in the comparison of RT between groups [post hoc on mean RT and t-test on differences in RT: t(33) = 1.74, p = 0.04; Figure 4 A1, lower part]. We also observed (3-way ANOVA; factors group, task, arm) that the decrease in RT corresponded to a rise in RT variability [RT standard deviation (RT_SD) Figure 4 B1] from the GOT to CMT [main effect task: F(1,33) = 14.52, p = 0.001] in FCb patients but not controls [main effect group: F(1,33) = 4.25, p = 0.04; interaction task × group F(1,33) = 5.59, p = 0.02; post hoc FCb GOT vs FCb CMT: p = 0.001; post hoc control GOT vs control CMT: p = 0.57]. Accordingly, the differences in RT_SD was higher for FCb subjects than controls [Figure 4 B1, lower part; t-test: t(33) = 2.37, p = 0.01].

In our analysis of trial sequences (Figure 4 C1), after a stop trial, RT-slowing was evident in controls and FCb patients. In both groups, the RT in the post-stop_correct_ (postC) and post-stop_error_ (postE) trials increased significantly (higher than 0; t-test all ps<0.05). By 3-way ANOVA (factors: group, trial type, arm), controls and FCb patients slowed their RT after erroneous stop trials more than after correct stop trials [main effect trial: F(1,33) = 18.90, p = 0.001; post hoc Control post-stop_correct_ vs Control post-stop_error_: p = 0.01; post hoc FCb post-stop_correct_ vs FCb post-stop_error_: p = 0.001]. FCb patients had a longer and significant post-stop_error_ slowing than controls [main factor group: F(1,33) = 8.73, p = 0.006; post hoc FCb post-stop_error_ vs Control post-stop_error_: p = 0.02]. The difference in RT for post-stop_correct_ trials did not differ between groups (post hoc FCb post-stop_correct_ vs Control post-stop_correct_: p = 0.24).

**Figure 5:**
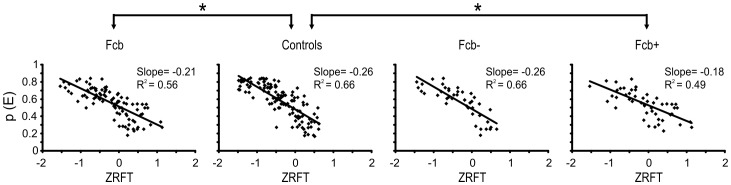
Normalized inhibition function. Each plot shows the group's probability to fail in canceling the stop trials (pE) as a function of ZRFT (black dots). The goodness of fit, slopes of the regression lines (black solid lines), and significant differences (*; p<0.05) between groups are indicated.

The duration of the stop process (SSRT; see Methods) in the FCb group did not differ from that of controls [Table 5; two-way ANOVA; factors: group, arm; factor group: F (1,33) = 0.25, p = 0.62]. Between arms, SSRT did not differ in any group [main factor arm: F(1,33) = 0.02, p = 0.89; interaction F(1,33) = 0.11, p = 0.75]. Conversely, the slope of the regression line of the normalized inhibition function (ZRFT) in FCb subjects was significantly lower compared with controls [ANCOVA and post hoc comparisons: F(1,219) = 4.23; p = 0.04; see Figure 5 for details].

We also explored the relationship of the motor score (see Methods) with the go process and the stop process duration. The RT of FCb patients in the two tasks significantly correlated with the motor score (GOT: r = 0.83, p = 0.001; CMT: r = 0.57, p = 0.04) suggesting a relationship between RT and motor deficits as measured by the modified version of the cerebellar motor deficit scale. Conversely, patients' SSRT did not correlate with either motor score (r = 0.36; p = 0.23) or post-error slowing (r = 0.52; p = 0.07), indicating a lack of relationship between motor deficits and executive functions as measured by the countermanding task.

Overall, FCb subjects had a longer go process than controls in the GOT and CMT. The go process on the CMT was particularly affected in FCb patients: the slowness of the response, due to changes in the behavioral context, on CMT was accompanied by a significant increase in variability. In the analysis of trial history effects, the increase in RT in FCb patients was longer after errors. FCb patients experienced deficits in responding to the stop signal–i.e., in triggering the stop process (as estimated by ZRFT regression line slope)– although the time to complete it (as estimated by the SSRT), once started, did not differ from controls.

### Cerebellar patients are more compromised when the lesion includes the DCN

The shaded grey areas in Figure 4 (panels A2, B2, C2) show the performance characteristics for FCb subjects, grouped by involvement (FCb+) or noninvolvement (FCb−) of DCN (see also Table 4 and 5) by the lesion. Statistical results refer to the analysis of these data and Controls data, as reported in Figure 4 A1, B1, C1.

By 3-way ANOVA (factor: group, task, arm; Figure 4 A2), group [F(2,32) = 14.32, p = 0.001] and task [F(1,32) = 22.25, p = 0.001] were significant main factors with regard to RT. The main effect of arm was not significant [F(1,32) = 3.16, p = 0.08]. Post hoc comparisons demonstrated significant differences in RT between GOT and CMT trials in each group (GOT RT vs CMT RT: Controls, p = 0.001; FCb+, p = 0.02; FCb−, p = 0.049). The between-group post hoc analysis also revealed significant differences in RT on the CMT between FCb+ and FCb− and Controls (FCb+ vs Controls, p = 0.001; FCb− vs Controls, p = 0.006). In contrast, between-group differences in RT on the GOT were not detected (GOT RT: Controls vs FCb+, p = 0.06; Controls vs FCb−, p = 0.05; FCb+ vs FCb−, p = 0.78).

By 3-way ANOVA of RT_SD (Figure 4 B2), we observed a significant effect of the main factor task [F(1,32) = 16.16, p = 0.001] and the group × task interaction [F(2,32) = 4.04, p = 0.03]. Significant differences were noted between tasks in FCb+ (p = 0.001) but not FCb− or Controls (GOT RT_SD vs CMT RT_SD: Controls, p = 0.24; FCb−, p = 0.12). No main effect of group or arm was detected [group: F(2,32) = 2.46, p = 0.10; arm: F(1,32) = 0.24, p = 0.62]. All other interactions between factors were not significant [interaction group × arm: F(2,32) = 2.46, p = 0.10; interaction task × arm: F(1,32) = 0.54, p = 0.47; interaction group × task × arm: F(2,32) = 2.26, p = 0.12].

Overall, our results suggest that when performing a movement implies a cognitive load, such as in CMT trials, cerebellar damage corresponds to a slowdown in readiness and an increase in the variability of responses to the Go signal. The same between-group differences did not appear in the GOT trials. Notably, between the GOT and CMT with regard to the RT and RT_SD, FCb+ patients performed worst. They had the largest increases in RT and RT variability, differing significantly from Controls in GOT when compared to CMT [One-way ANOVA (F (1,32) = 22.25, p = 0.001; post hoc p = 0.04] and in RT_SD difference [One-way ANOVA F (1,32) = 16.16, p = 0.001; post hoc p = 0.008] (Figure 4, bottom part of A2 and B2, respectively, compared with Controls in A1 and B1). The increase in RT and RT_SD between the CMT and GOT did not differ between Controls and FCb− (post hoc RT difference, p = 0.55; post hoc RT_SD difference, p = 0.40).

FCb+ patients also had the largest post-error slowing (Figure 4, panel C2 compared with Controls in C1). By 3-way ANOVA (factors: group, trial type, arm), there was a significant main effect of group [F(2,32) = 5.21, p = 0.01] and trial type [F(1,32) = 17.37, p = 0.001]. In post hoc comparisons, FCb+, FCb−, and controls had significantly longer post-stop_error_ slowing than post-stop_correct_ slowing (post-stop_error_ vs post-stop_correct_: Controls, p = 0.018; FCb−, p = 0.013; FCb+, p = 0.03), and FCb+ post-stop_error_ slowing was significantly longer than in controls (p = 0.02). FCb+ and controls did not differ significantly with regard to post-stop_correct_ slowing (p = 0.06). FCb− did not differ from controls in post-stop_error_ (p = 0.46) or post-stop_correct_ slowing (LSD Fisher, p = 0.32). Significant differences in post-stop_error_ (LSD Fisher, p = 0.15) and post-stop_correct_ slowing (LSD Fisher, p = 0.56) were not observed between FCb+ and FCb− patients. The main effects of arm [F(1,32) = 0.55, p = 0.46] and its interactions were not significant [interaction arm × trial: F(1,32) = 0.32, p = 0.57; interaction arm × group: F(2,32) = 0.35, p = 0.71; arm × group × trial: F(2,32) = 2.56, p = 0.08].

Stop process duration, measured as the length of the SSRT, did not differ between controls and patients or between the FCb+ and FCb− subgroups [2-way ANOVA; factors: groups, arm; factor group: F(2,32) = 1.73, p = 0.19; factor hand: F(1,32) = 0.01, p = 0.93; interaction: F(2,32) = 1.26, p = 0.30]. Conversely, the slope of the ZRFT, indicative of stop trigger efficiency, resulted shallower in FCb+ but not FCb− subjects [ANCOVA F(2,217) = 3.57, p = 0.03 and post hoc comparisons: p<0.05; see Figure 5 for details].

Finally, we controlled for the effect of the side of the cerebellar lesion for all behavioral parameters. During the recruitment of patients with and without DCN involvement, FCb subjects were not well distributed with regard to the side of the lesion (Table 1; 8 and 5 patients had lesions primarily in the right [FCb_R] and left [FCb_L]– cerebellum, respectively). Nevertheless, we extended the analyses above to include lesion side as a factor. By three-way ANOVA (factors: lesion side, arm, task), there were no differences between FCb_L and FCb_R in performance on the GOT and CMT. Both groups of patients were slower than controls in performing both tasks [main effect lesion side: F(2,32) = 14.30, p = 0.001] and performed the CMT with a longer RT than on the GOT [main effect task: F(1,32) = 19.83, p = 0.001; interaction between task and lesion: F(2,32) = 2.15, p = 0.13]. No between-arm differences were observed [main effect arm: F(1,32) = 2.78, p = 0.13]. The only significant effect was an increase in the variability of RT in FCb patients with right lesions compared with controls (p = 0.03 post hoc; 3-way ANOVA; factors: group, task, arm) by contrasting RT on the GOT versus CMT [main effect lesion side: F(2,32) = 2.49, p = 0.10; main effect task: F(1,32) = 13.92, p = 0.001; main effect arm: F(1,32) = 0.31, p = 0.59].

No differences between FCb_L and FCb_R were detected in post-stop performance. By three-way ANOVA (factors: lesion side, hand, trial type), there was a general increase [main effect trial type: F(1,32) = 19.95, p = 0.001] and a significant difference between controls and patients [main effect lesion: F(2,32) = 4.45, p = 0.02] in post-stop_error_ slowing. No significant effects of arm [F(1,32) = 0.35, p = 0.35] or the interaction between trial type and lesion side on post-stop slowing were detected [F(2,32) = 1.77, p = 0.19]. By two-way ANOVA (factors: lesion side, arm), there were no significant differences in SSRT between lesion side [main effect lesion side: F(2,32) = 2.11, p = 0.14; main effect arm: F(1,32) = 0.0006, p = 0.98; interaction between lesion side and arm: F(2,32) = 0.07, p = 0.93]. Based on ZRFT, there was no difference in the slope between FCb_L and FCb_R [F(2,217) = 2.35, p = 0.10].

In conclusion, our cohort of focal cerebellar patients (FCb) have significant differences from controls in the go and stop processes when lesions involve the DCN. Although the FCb+ and FCb− subgroups had longer RTs overall than controls, the go process in the CMT was affected only in FCb+ patients, as evidenced by the significant increase in variability when the go signal was followed by a stop signal. In our analysis of the trial history effect, the increase in RT after errors was more evident in FCb+ subjects. Despite having comparable SSRTs as controls, FCb+ subjects, but not FCb−, were impaired in their ability to trigger the stop process.

## Discussion

We report the performance of FCb subjects on a CMT and provide evidence that the cerebellum has a specific function in the cognitive control of movement suppression and performance monitoring, two core properties of EC [2,25].

### Cerebellar function in executive motor control

According to evidence in humans and animals [3,4,25], various areas of the frontal cortex and subcortical structures contribute to the voluntary control of movement. Among subcortical structures, the subthalamic nucleus (STN) is often proposed as a significant area in the basal ganglia that supports the cortical control of the generation of movements by gating the inhibitory control of the internal segment of the globus pallidus (GPi) over the thalamic drive [7,10].

The STN is linked functionally to the inferior frontal cortex (IFC), presupplementary motor areas (preSMAs), anterior cingulate cortex (ACC), dorsal premotor cortex (PMd), and frontal eye field (FEF)–areas that regulate the executive control of movement generation 10,25,55–58. With regard to motor control, basal ganglia models [59–62] suggest that movement can be suppressed by activation of the STN through the ‘hyperdirect’ (in which the STN receives a direct excitatory projection from the cortex) or ‘indirect’ (the STN is activated by contrasting–via the striatum–the inhibition that is exerted by the external segment of the globus pallidus [GPe]) pathway.

The use of neurotrophic viruses to track the connections between areas of the brain has provided anatomical evidence for the reciprocal influence of the cerebellum and cortical-basal ganglia-cortical loops, wherein DCN, the output of the cerebellum, influence the ‘indirect’ pathway by specifically targeting striatal neurons that project to the GPe; in turn, the STN projects to pontine nuclei and thus governs the activity of the cerebellar cortex [12]. The emerging model contends that frontal cortical areas (e.g., the PFc, SMA, and PMd) send movement-related decisions to the cerebellum and basal ganglia. Both nodes send signals back to the primary motor cortex via the thalamus that are sufficient to permit or facilitate execution of the programmed movement or, conversely, countermand it by activating the indirect or hyperdirect pathway [62]. The interplay between the cerebellum and basal ganglia could thus contribute to fine executive control of motor generation.

Our results show that FCb patients do not experience deficits in the duration of the stop process. The SSRT was similar between cerebellar and control subjects. Grouping the patients by DCN involvement did not yield further information. However, the comparison of ZRFT slopes suggests that FCb patients have specific inhibitory deficits, failing more often than controls in triggering the stop process. In our group of patients, the impairment was evident when the lesions involved the DCN. In FCb+ patients, the slope of the inhibition function was the shallowest measured out of the slope measured in the other groups and the only one that differed significantly from slope in controls.

In our analysis of the go process, patients took longer than controls to react to the go signal. Prolonged RT is a hallmark of cerebellar patients [63]. Luciani [64] proposed that DCN exert a tonic facilitatory influence over the motor cortex. The activity of DCN is in turn modified by the inhibitory control of the cerebellar cortex [65]. We observed that this effect correlates with the cognitive load of the motor task that is performed–e.g., when a sudden stop signal is expected in a sequence of standard reaction time trials, requiring cancellation of the action. This form of control is commonly termed proactive control [52].

We propose that the cerebellum receives a copy of the cortically originated signal to suppress a movement via the STN [10,12] and contributes to executive control by modulating the tonic inhibition that is exerted by the cerebellar cortex on the DCN. The results of this process are normally relayed back to the basal ganglia through the indirect pathway, influencing its contribution to inhibitory action. Our results suggest that focal lesions preserve the contribution of the cerebellum in determining the speed of the stop process. Action interruption occurs with lower probability in patients, but when it does occur, it takes the same amount of time as controls; this was particularly evident after DCN damage.

However, our results could have been influenced by the small number of subjects in the FCb subgroups after sorting patients by DNC involvement, which could have introduced effects of other factors that we did not control for with regard to the behavioral performance of FCb patients. For example, as shown in Table 2, FCb+ and FCb− did not match controls for the gender and had a nonhomogeneous distribution of it. Recent evidence has highlighted a disparate pattern of activation of cerebral areas between males and females during the execution of stop trials in the CMT [66,67]. However, this gender effect was not affecting the reported performance on the task. We observed only a significantly longer post-stop_error_ slowing in female than male controls (see Tables 4 and 5; statistical details are described in the caption), contrasting what we observed on grouping DCN lesions, because the FCb+ group comprised primarily males (Table 2).

Similarly, no main effect of the lesion side was detected, as described in other motor paradigms that are focused on procedural learning and time elaboration [68,69] and in neuroimaging studies [70,71]. However, due to the difference in the number of subjects in the FCb_R and FCb_L groups (see Table 2) we cannot exclude the possibility that the absence of a lesion side effect was related to the difference in sample size. These aspects require further investigation in future studies.

### Cerebellar function in the proactive control of behavior and error monitoring

In the CMT, go trials always have the potential to turn into stop trials, thus requiring a continuous update (lengthening) of the RT to maximize stop success [2,50]. We observed that the RT of go trials in the CMT was significantly longer than in the GOT for the patient and control groups, more so in the former. Cerebellar patients were strongly affected by the trial history effect. Their increase in go trial RT, following a stop trial, was larger than in controls, especially after having failed to stop (i.e., they experienced notable post-error slowing). This deceleration became significant if the lesion involved the DCN, indicating that in performing a basic task (*go trials*), these patients experienced greater perturbations by an infrequent and unpredictable event (*stop trial*) and by errors than other subjects.

This evidence also supports the hypothesis that the anatomical link between the basal ganglia and cerebellum has a significant function in executive control. Our data suggest that the contribution of the basal ganglia to error-related cognitive control, as reported [16–19,72], requires efficient interaction with the cerebellum.

The cerebellum has been hypothesized to mediate the correction of errors in the motor and cognitive domains [73–77]. The cerebellum, in a network that comprises the PFC, the dorsal ACC, thalamus, and SMA, has also been proposed to affect behavioral adjustments after presentation of a stop signal in the CMT [11]. Our data provide evidence that a lesion in the cerebellum impairs the processing of errors and that this effect is present when DCN are involved.

Notably, the effects of DCN damage that we observed are consistent with previous studies on various cognitive domains [28,78,79]. However, our findings must be confirmed in future studies using advanced methods to better characterize the site of the lesion in the DCN [80]. Such steps will allow us to determine the various functions of the interposite and dentate nuclei in cerebellar-cortical loops.

Moreover, future studies should focus on assessing the CMT in patients who are affected by degenerative cerebellar atrophy, in which the Purkinje cells are more selectively affected and thus influence the inhibitory control of DCN.

In conclusion, our study has provided evidence of impaired efficiency of execution of actions and performance monitoring in the presence of cerebellar damage, increasing our understanding of motor and cognitive cerebellar symptoms and the consequent behavioural impairments. We propose that the cerebellar function in executive control is mediated by its reciprocal interaction with the basal ganglia, although the possibility of a direct cerebello-prefrontal interaction cannot be excluded in controlling the evaluated functions.
